# A high-throughput core sampling device for the evaluation of maize stalk composition

**DOI:** 10.1186/1754-6834-5-27

**Published:** 2012-05-01

**Authors:** German Muttoni, James M Johnson, Nicholas Santoro, Craig J Rhiner, Karl J Haro von Mogel, Shawn M Kaeppler, Natalia de Leon

**Affiliations:** 1 Department of Agronomy, University of Wisconsin, 1575 Linden Drive, Madison, WI, 53706, USA; 2 DOE Great Lakes Bioenergy Research Center, Michigan State University, 164 Food Safety and Toxicology Building, East Lansing, MI, 48824, USA; 3 Physical Plant, University of Wisconsin, 1217 University Avenue, Madison, WI, 53706, USA; 4 DOE Great Lakes Bioenergy Research Center, University of Wisconsin, 1575 Linden Drive, Madison, WI, 53706, USA

**Keywords:** Biofeedstock, Maize, Cell wall composition, High-throughput stalk sampling

## Abstract

**Background:**

A major challenge in the identification and development of superior feedstocks for the production of second generation biofuels is the rapid assessment of biomass composition in a large number of samples. Currently, highly accurate and precise robotic analysis systems are available for the evaluation of biomass composition, on a large number of samples, with a variety of pretreatments. However, the lack of an inexpensive and high-throughput process for large scale sampling of biomass resources is still an important limiting factor. Our goal was to develop a simple mechanical maize stalk core sampling device that can be utilized to collect uniform samples of a dimension compatible with robotic processing and analysis, while allowing the collection of hundreds to thousands of samples per day.

**Results:**

We have developed a core sampling device (CSD) to collect maize stalk samples compatible with robotic processing and analysis. The CSD facilitates the collection of thousands of uniform tissue cores consistent with high-throughput analysis required for breeding, genetics, and production studies. With a single CSD operated by one person with minimal training, more than 1,000 biomass samples were obtained in an eight-hour period. One of the main advantages of using cores is the high level of homogeneity of the samples obtained and the minimal opportunity for sample contamination. In addition, the samples obtained with the CSD can be placed directly into a bath of ice, dry ice, or liquid nitrogen maintaining the composition of the biomass sample for relatively long periods of time.

**Conclusions:**

The CSD has been demonstrated to successfully produce homogeneous stalk core samples in a repeatable manner with a throughput substantially superior to the currently available sampling methods. Given the variety of maize developmental stages and the diversity of stalk diameter evaluated, it is expected that the CSD will have utility for other bioenergy crops as well.

## Background

Plant biomass yield and composition are key attributes in the development and utilization of crops for cellulosic biofuel [1,2] as well as other industrial uses. Compositional analysis of large numbers of plant biomass samples is needed to support breeding, genetics, and production research in large scale.

Currently, highly accurate, reproducible and precise robotic analysis systems are available for the evaluation of biomass composition on a large number of samples and using a variety of pretreatments [3,4]. However, the lack of an inexpensive and high-throughput process for large scale sampling of those biomass resources is still an important limiting factor. Therefore, the development of tools that allow high-throughput sample collection from biomass tissue in a highly repeatable manner is an important step in the development of efficient research platforms for bioenergy and other industrial applications.

In the case of maize, the standard sampling process for field scale plots involves: 1) harvesting the stover using a row-plot forage harvester or specifically designed customized research combine, 2) drying and grinding the stover samples, and 3) evaluating of composition of those samples [4-7]. Similar processes are used in other grasses and many forage legumes species. Maize researches may be interested in the overall composition of the stover or any of the particular plant fractions such as stalks, leaves, cobs, shanks and husks. For the latter, the different plant parts are typically manually dissected, dried, chopped and ground [8-11]. Whether the whole stover or particular plant fractions are evaluated, the approaches commonly used for sample collection and evaluation, are labor-intensive and, therefore, can only be done on a limited number of samples.

Maize cell wall composition and digestibility vary significantly across plant fractions [9,11-13] resulting in large heterogeneity within whole-plant (e.g. stover) samples. Several studies have demonstrated that the stalk (i.e. main stem) represents the highest proportion (46-53%) of the total plant dry biomass and the most recalcitrant component of the plant at grain physiological maturity, whereas the leaves represent 21-30% of the total dry biomass and are more digestible [9,11-13]. Given that maize stalks are the largest contributor to overall dry biomass, analysis of this plant fraction is expected to provide insights into cell wall composition and digestibility of the whole plant stover.

Our goal was to develop a simple mechanical stalk core sampling device that could be used to collect uniform samples of a dimension compatible with robotic processing and analysis, while allowing the collection of hundreds to thousands of samples per day. We optimized our tissue core sampling device (CSD) for collection of maize stalks in a format compatible with a high-throughput digestibility platform (HTDP) previously described [3].

## Results and discussion

### Core sampling device and sampling procedure

**Figure 1:**
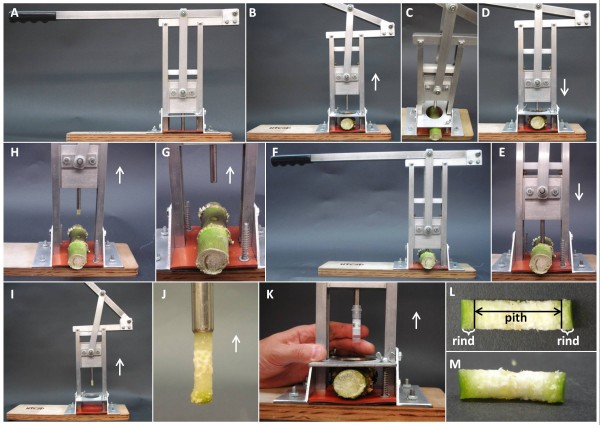
**Core sampling device.** Core sampling device (CSD) (**A**). The CSD handle is lifted-up (the white arrow indicates the direction of the handle) and the maize main stalk internode is placed on the rubber mat (**B** and **C**). Once the internode is in the right position (center of rubber mat), the handle is pulled down (**D**) and the cork borer begins to penetrate the internode (**E**) until both sides of the internode (rind) are cut (**F**). The handle is lifted up again (**G**) and the core is released by a core extractor mechanism (**H**, **I**, and **J**). A tube is placed over the end of the cork borer before the sample is released (**K**). The sample consists of a complete cross-section of the stalk internode composed of both rind sections as well as the pith (**L**, **M**). Note: in E-H, the separation plate was removed to facilitate visualization.

**Figure 2:**
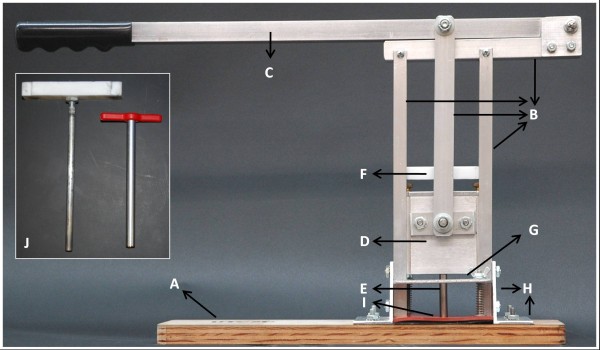
**Core sampling device components.** The core sampling device (CSD) is composed of nine main components that include a platform (**A**), frame composed by the arms and top bar (**B**); handle (**C**); cork borer housing (**D**), cork borer (**E**); core sample extractor (**F**); separation plate (**G**); two base brackets (**H**) and a rubber mat (**I**). The core sample extractor and the core borer are shown individually (**J**).

We have developed a simple, robust core sampling device (CSD) for the collection of tissue cores from plants (Figure 1). A CSD unit can be operated by one person without the need for prior training or specific expertise. The CSD is constructed primarily from aluminum components and contains replaceable nylon bushings at the friction points and adjustable wing nuts (Figure 2). Several features were incorporated to enhance the throughput and reproducibility of the sampling process while minimizing the manipulation of samples that can result in errors. Among these key features is the core sample extractor (Figure 2F) that fits within the cork borer (Figure 2E) and enables the complete removal of the sample while keeping the sectioned core intact (Figure 1L and M). Extraction of the core from the cork borer begins once the extractor head reaches the frame top bar (Figure 2B) and as the handle is lifted (Figure 1H and I). Immediately before extraction of the core is initiated, a tube is placed in the cork borer and the sample is released inside the tube avoiding the exposure of the core to touching or other sources of contamination. This is an important feature of the CSD as it eliminates the need to clean out the cork borer between consecutive cores and produces a sample that is free of contamination from prior core sections.

A tissue core sample can be obtained in about 5–10 seconds from the time when the sample (e.g. internode) is placed in the CSD until the core sample is released inside a tube (Figures 1B through 1K). In addition, another very useful and convenient feature of the CSD is that once a sample is released into a tube, it can be placed directly into a bath of ice, dry ice, or liquid nitrogen maintaining the composition of the biomass sample for relatively long periods of time.

**Figure 3:**
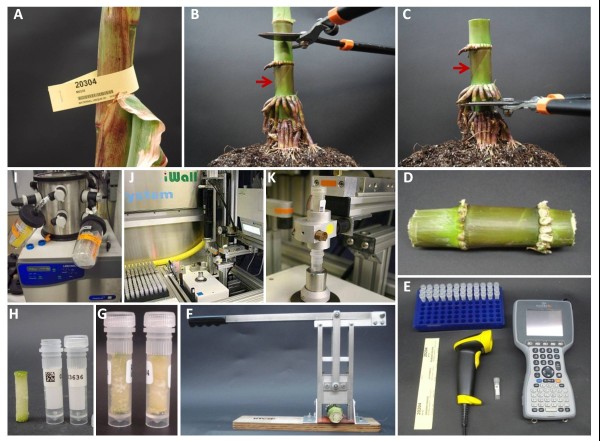
**Core sampling procedure**. The process of obtaining core samples involves the identification of a sample (e.g. plant, plant fraction or field plot) using a barcode row-tag (**A**). In this example, the second internode above-ground (red arrow) is obtained by cutting the third (**B**) and first (**C**) internodes above ground (**D**). The process requires reading (with a barcode scanner) and recording (with a field computer) the barcode tag and barcode tube or tubes, if multiple samples are collected (**E**). The core sampling device (**F**) is used to obtain the stalk core samples (**G**, **H**). The tubes with a perforated screw cap and the sample are dried using a freeze drier (**I**) and then analyzed in the high-throughput digestibility platform (**J**, **K**).

In our use of the CSD to sample maize internodes, the device was integrated into a sampling procedure that resulted in an overall throughput of up to 1,000 core samples in an eight hour period (Figure 3). Key features of the integrated procedure include the use of a) barcode labels to identify the sample unit (e.g. individual plant, plant component or field plot), and the tube, b) a scanner used to scan the barcoded sample label and the tube label, c) a field computer or laptop computer connected to the barcode scanner for electronic storage of the sample and tube information (Figure 3E). Once samples are collected, they are lyophilized inside the tubes with perforated caps (to allow moisture flow) for at least 72 h (Figure 3I). In this example, the samples are then robotically ground and evaluated biochemically for cell wall recalcitrance as described by Santoro et al. [3]. A supplemental movie file shows the use of the CSD and the sampling procedure in more detail (Additional file 1). Additionally, a supplemental computer-aided design (CAD) file shows the single individual components of the CSD and their specific dimensions (Additional file 2).

More than 17,000 maize stalk core samples were collected in the field in the fall of 2011 using the CSD. During the collection of these samples none of the structural components needed replacement. We designed the CSD to allow replacement of the parts expected to experience the most wear – the cork borer and the rubber mat that the cutter presses against. After 1500–2000 samples the cork borer and the rubber mat were replaced (Figure 2F, I, and J). The replacement of these two components can be done in 5–10 minutes by hand, without requiring tools.

### Variation across internodes within a plant

**Figure 4:**
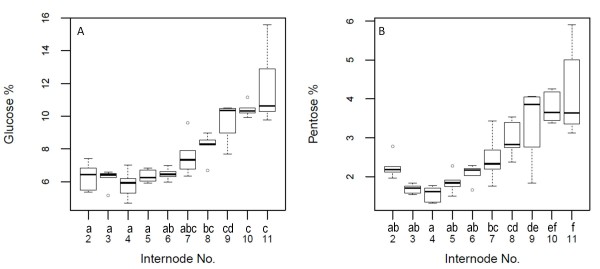
**Box-plots of across internode variability for glucose (A) and pentose (B) yield.** Cell wall glucose and pentose yield (%) were measured from the second to the eleventh main stalk internodes above-ground. A total of five plants from maize inbred line B73 grown in the glasshouse were measured for each internode. Letters above the internodes numbers (x-axis) corresponds to the mean comparisons adjusted for multiple testing with Bonferroni method. Internodes that share a letter are not significantly different at *p* = 0.05.

We evaluated the utility of the CSD to assess the cell wall recalcitrance of maize inbred lines across internodes within a plant and compared the results with those found in previous studies. The two traits measured were glucose and pentose yield using the HTDP [3]. Above-ground internodes two to eleven were sampled from plants of the B73 maize inbred line grown in a glasshouse. We found differences in both glucose and pentose yield (p < 0.001) among internode positions (Figure 4). For both glucose and pentose yield, the digestibility from lower to upper internodes followed an exponential trend with digestibility being greater in the upper internodes. Interestingly, cell wall glucose yield measured in internodes two through six was significantly different from that obtained in internodes nine through eleven according to the multiple means comparison (Figure 4A). In the case of pentose yield, internodes two through six were different from internodes eight to eleven (Figure 4B). Furthermore, the variability within the lower internodes was smaller than that observed in upper internodes. According to Jung et al. [14], upper internodes of maize, (when evaluated at the 15th-leaf stage or V15), are comprised of undifferentiated cells undergoing elongation whereas, lower internodes are fully elongated and engaged in secondary cell wall development. It has also been shown that lignin concentration decreases from lower to upper internodes [15]. Both studies [14,15] showed that glucose, xylose and total cell wall digestibility increase from lower to upper internodes following a trend similar to what is observed here. These results suggest that sampling at lower internodes provide a more consistent measurement of glucose and pentose yield across developmental stages. Based on these results we decided to do further analysis utilizing the second elongated internode above ground.

### Variation within the second elongated internode

We were interested in determining the range of variability that exists among samples taken at different positions within a single maize internode. To this end, three relative positions within an internode, identified as bottom, middle, and upper sections, were evaluated in four maize inbred lines (NK794, MS116, DKPB80 and A208). A weak statistical difference for the effect of sampling position within an internode was observed for both glucose and pentose yield (p = 0.08 and p = 0.10, respectively) (see Additional file 3: Table S 1 and Additional file 4: Table S 2). As expected, due to the diverse nature of the germplasm sources used, significant variation was observed among maize lines for glucose and pentose yield. Even though there was a weak evidence for a sampling position effect, the difference in glucose and pentose yield means and standard deviations were not substantially large (Table 1). Therefore, taking a single core sample within the center of an internode is a practically viable approach to sample biomass composition and allows the detection of differences among maize lines.

**Table 1:** Means and standard deviations of glucose and pentose measured at multiple positions within an internode

	**Glucose yield**			**Pentose yield**		
**Position**	**Bottom**	**Middle**	**Upper**	**Bottom**	**Middle**	**Upper**
**Mean (%)**	8.89	8.55	7.80	3.74	4.23	3.68
**Standard deviation (%)**	1.07	1.52	1.09	0.71	0.85	0.90

Glucose and pentose yield were measured in three relative positions (bottom, middle and upper sections) of the second internode of three plants for each of four diverse maize inbred lines. The mean of the three plants was used to calculate the standard deviations.

### Correlation of repeated measures across time

**Figure 5:**
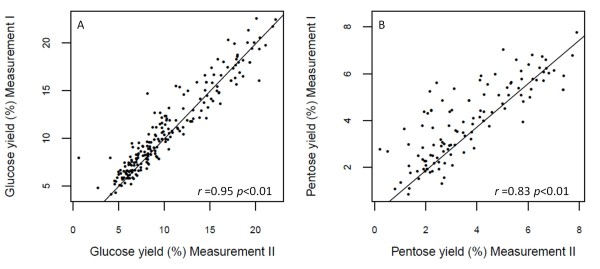
**Pearson’s correlation (*r*) between repeated measurements of cell wall glucose (A) and pentose yield (B).** Cell wall glucose and pentose yield (%) correlation between two measurements taken at two different times (eight months apart) was estimated using 211 and 117 samples, respectively.

Repeatability, defined as the correlation between repeated measurements over time in the same individual [16] is a useful parameter to assess the level of variability involved in the collection of core samples and in the HTDP analysis. The Pearson’s product–moment correlation between the two repeated technical measurements in a set of 211 and 117 randomly selected samples taken eight months apart was 0.95 (p < 0.001) and 0.83 (*p* < 0.001) for glucose and pentose yield, respectively (Figure 5). These very high correlations indicate that there is relatively little technical error among epeated samples subjected to the HTDP analyses and that several sub-samples yield consistent and representative results. This high level of precision implies that only a single sample would be needed to obtain reliable results.

### Effect of storing internodes at low temperatures

**Figure 6:**
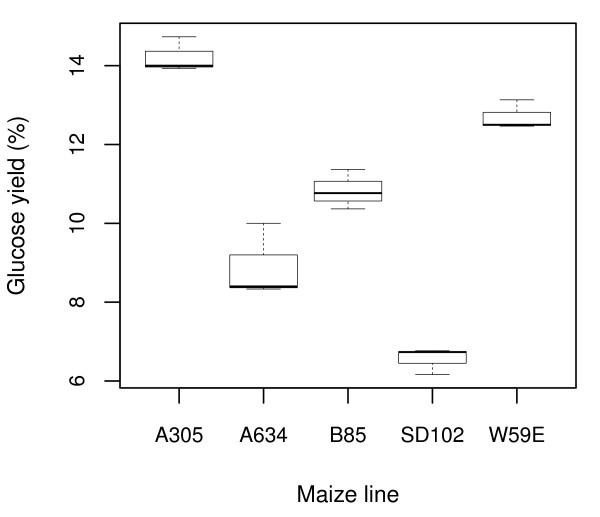
**Box-plots of cell wall glucose yield for five maize inbred lines**. Cell wall glucose yield (%) was measured at three different times (0, 24 and 60 h) in the second internode above ground from three different plants in five diverse maize inbred lines. The mean of the three plants was used to plot the variability of each inbred line.

There are circumstances in which it would be convenient to harvest stalk material and then proceed with the isolation of cores at a later time. To determine the effect of storage before core sampling, we evaluated stalk cores from five maize inbred lines (A305, A634, B85, SD102 and W59E), extracted after internodes were stored at ~4°C for 0, 24 and 60 hours. The analysis of variance indicates that there is no effect of storage (p > 0.40) on cell wall glucose yield among the five maize lines (Additional file 5: Table S 3) within the time-frame evaluated, whereas significant variation (p < 0.001) was observed among the five inbred lines tested (Figure 6, Table 2, Additional file 5: Table S 3). These results indicate that internodes can be stored at low temperatures (~4°C) for as long as 60 hours without altering the measurement of cell wall glucose yield and allowing the detection of genotype differences (Table 2).

**Table 2:** Glucose means and least significant differences for five maize inbred lines

**Maize inbred line**	**Glucose yield (%)**
A305	14.22 *a*
W59E	12.70 *b*
B85	10.83 *c*
A634	8.91 *d*
SD102	6.56 *e*
*LSD (α = 0.05)*	*1.06*

Cell wall glucose yield (%) was measured at three different times (0, 24 and 60 h) in the second internode above ground from three different plants in five diverse maize inbred lines. The analysis of variance was performed on the mean of the three plants. The least square difference (LSD) corresponds to the Fisher's protected LSD*.* Means with the same letter are not significantly different.

### Advantages of using core samples

The high technical repeatability, sample homogeneity and substantial superior throughput support the use of the CSD for biomass composition analysis in maize. One of the main advantages of using cores compared to whole-plant samples, such as maize stover or forage samples is the high level of homogeneity of the sample. In this study, using maize internodes, the samples are composed entirely by the stem tissues (rind and pith) sampled at a constant position (i.e. second internode above ground). Maize stover is a mix of multiple plant tissues and organs (stalks, leaves, husks, shanks and cobs) that are known to have very different cell wall composition and digestibility [9,12]. Current methods harvest whole plant samples with a row-plot forage harvester or specifically designed customized research combine, and these samples represent the product that will likely be utilized in large scale biorefineries. Alternatively, if specific plant fractions are of interest, manual plant dissection and sampling is required [8-11]. Both of these approaches are labor-intensive and, therefore, can only be done on a limited number of samples (in the order of hundreds of samples a day). When the goal is to evaluate single plant fractions, the CSD produces core samples that are very well suited for biochemical analysis because they represent a homogenous and reproducible sample suitable for high-throughput screening platforms that require only few milligrams of biomass. In the particular case of maize, given that the stalk is the largest fraction of total maize dry biomass at physiological maturity [9,11-13], one would expect the results obtained with the maize internode cores to be representative of the individual main component of maize stover. Thus, this information should assist in the identification of extremes in the distribution of biomass composition in a given maize germplasm.

### Use of the core sampling device in other plant species

Even though the CSD was tested exclusively using maize stalks, this device has the potential to be used in other species and tissues in which cores can be obtained (e.g. sorghum, *Mischanthus*, etc.). The cork borer utilized to sample maize stalks had a diameter of 7.8 mm. Nevertheless, cork borers of 4.8-7.8 mm (available form several laboratory suppliers) can be utilized. We have tested the CSD across a range of physiological maturities from plants that were at the onset of grain filling (R2 or the blister stage, about 10–14 days after silking [17]) to plants that were at grain physiological maturity (R6, 55–65 days after silking [17]). Also the samples represented a range of stalk density and stalk diameters (from 8 mm to 45 mm; data not shown). Hence, our experience with variable maize genotypes indicates that the CSD is potentially well suited for other bioenergy crops such as sorghum or *Miscanthus*.

## Conclusions

The device that we have developed has been demonstrated to successfully produce homogeneous maize stalk core samples in a repeatable manner with a throughput substantially superior to the currently available sampling methods. With a single core sampling device operated by one person with minimal training, more than 1,000 biomass samples can be obtained in an eight-hour period. One of the main advantages of using cores is the high level of homogeneity of the samples obtained and the minimal opportunity for sample contamination.

## Materials and methods

### Variation across internodes within a plant

Five plants of the maize inbred line B73 grown in a glasshouse (16:8 hours of light:dark photoperiod, 28/20°C day/night temperatures) were sampled from the second to the eleventh internode above ground. The five plants were grown in the glasshouse for 90 days before being sampled. The analysis of variance and means comparison were performed using the R software [18]. Cell wall glucose and pentose yield (%) were measured in internodes two to eleven.

### Effect of storing internodes at low temperatures

Five maize inbred lines A305, A634, B85, SD102 and W59E were randomly chosen from a set of 513 lines of the Wisconsin Diverse population [19]. The 513 lines were grown in a randomized complete block design (RCBD) with two replications. The experiment was planted in May 2010 in two row-plots 6 m in length and 0.76 m row spacing. The field experiment was conducted at the Arlington Agricultural Research Station, Madison, WI. The second elongated internode above ground was cut in each of three plants from the middle of the row for each maize line 45 days after female flowering (i.e. exposure of silks in 50% of the plants in a plot). This time of sampling approximately corresponds to grain physiological maturity stage or reproductive stage R6 [17]. The internodes were maintained in a regular refrigerator at a ~4°C during few minutes (0 h treatment), 24 and 60 h after field harvested. The analysis of variance was performed in the mean of the three plants of each row-plot using the R software [18]. Cell wall glucose yield (%) was measured in each internode. Least significant difference (LSD) was calculated using PROC GLM of the SAS® software version 9.2. The calculated LSD corresponds to the Fisher's protected LSD.

### Variation within the second elongated internode

Four maize inbred lines NK794, MS116, DKPB80 and A208 were randomly chosen from the same population described in the previous section. For each of these four lines, three plants were sampled in three positions, bottom (~0.5 cm above the lower node), middle (middle between the two nodes) and upper (~0.5 cm below the upper node) section of the second elongated internode above ground. The plots were harvested 45 days after silking and internodes stored for 24 h. Cell wall glucose and pentose yield (%) were measured in each internode at each position. The analysis of variance was performed in the mean of the three plants of each row-plot using the R software [18].

### Correlation of repeated measures across time

The correlation between two independent repeated measurements (technical repeatability) of glucose and pentose yield (%) was calculated using the R software [18]. The second measurement was taken eight months after the first measurement. A total of 211 and 117 randomly chosen samples were analyzed for glucose and pentose yield, respectively. These samples were part of the 513 lines grown in the field in 2010 (see “Effect of storing internodes at low temperatures” in the current section).

### High-throughput digestibility platform: grinding and dispensing samples

Cores samples were ground, fed, and weighed using the iWALL custom-designed robot (Labman Automation Ltd., United Kingdom). Between 60–100 mg of dried biomass were loaded with the CSD into Sarstedt 2-ml screw-cap microtubes (72.694 Sarstedt, Newton, NC) along with three 7/32 inch (5.56 mm) stainless steel balls (Salem Specialty Ball Co, Canton, CT). The tubes were placed into racks and positioned in the iWALL robot, and pulverization of the biomass was accomplished by ball milling. The grind time was sufficient to reduce the initial sample to a powder. At this point, samples are dispensed into 1.4-mL 2D Tracker U bottom Micronic tubes (MP52607PK, Micronic North America, McMurray, PA) that were scanned and weighed on iWALL. The target value was 1.5 mg per tube with a tolerance of ±0.2 mg and the precise weight of dispensed material was stored digitally. Samples were weighed and dispensed in triplicate. The tubes, along with assay controls, were arranged into Stabo-Racks (Micronic North America) and now ready for pretreatment. A detailed implementation of the iWALL system can be found in Santoro et al. [3].

### High-throughput digestibility platform: pretreatment and enzyme digestion

The racks were placed onto a PerkinElmer (Waltham, MA) Janus automated workstation and 750 μL of pretreatment solution was pipetted into each tube. The pretreatment solution consisted of 0.25% (wt/vol) NaOH (6.25 mM). Sample plates were sealed with a thermoplastic elastomer cap mat (# MP53000, Micronic North America), incubated at 90°C for 3 h in a water bath, and cooled on ice. Where necessary, reactions were neutralized with acid (HCl). Next, 50 μL of a solution containing 0.5 μL Accellerase 1000 (Genencor, Rochester, NY), 1 M citrate buffer (pH 4.5) plus 0.01% sodium azide was added to all tubes. Enzymatic hydrolysis was done in a final volume of 0.8 ml using an enzyme concentration of 50 mg protein/g glucan. Racks were incubated in a hybridization rotisserie oven (VWR, Model 5420) at 50°C for 20 h with end-over-end rotation. Following incubation the racks were centrifuged at 1,500 × g for 3 min in a swinging bucket centrifuge (Eppendorf, Germany, Model 5417R) and the supernatants were transferred into 0.8 mL deep-well 96-well plates. The liquids were then pipetted in quadruplicate into two 384-well plates; one plate was used for measuring glucose and the other for pentose.

### High-throughput digestibility platform: colorimetric assays for glucose and pentose

Glucose content of samples was assayed using the glucose oxidase/peroxidase (GOPOD) method (K-GLUC, Megazyme, Ireland). The assay volumes were reduced to 4 μL of the digestion reaction supernatant and 64 μL of the GOPOD reagent. This allowed the procedure to be performed in 384-well microtiter plates. The incubation times and temperatures followed the manufacturer’s instructions. This assay was also “miniaturized” with the volumes reduced to dispensing 12 μL sample and 56 μL p-bromoaniline in thiourea into 384-well microtiter plates. The samples were first incubated at room temperature for 70 min. Next, the plates were read at 520 nm, and the absorbance recorded (Abs1). Plates were heated at 100°C for 10 min, incubated a second time at room temperature for 70 min and a second absorbance reading recorded (Abs2). Pentose concentration is determined by subtracting Abs1 from Abs2 as described elsewhere [20].

### High-throughput digestibility platform: data collection and analysis of digestibility results

Every microplate contained its own set of standards for generating a standard curve, which was used to determine the amount of glucose or pentose in each sample. Biomass from each sample tube was dispensed in triplicate into three independent output vials, and each digestion assay was quantified for glucose and pentose in quadruplicate. Thus, final values are the average of 12 measurements. Glucose and pentose yield is the amount of each sugar relative to the total dry mass initially dispensed into each tube (% of dry biomass).

## Abbreviations

CSD, Core sampling device; HTDP, High-throughput digestibility platform.

## Competing interests

The authors declare that they have no competing interests.

## Authors' contributions

GM conceived and designed the study, collected and processed the core samples, performed statistical analyses, drafted the manuscript and contributed to the design of the core sampling device. JMJ contributed to the design of the device and the study, collected and processed the core samples, assisted with manuscript writing, and performed statistical analyses. NS contributed to design of the core sampling device and the study, and processed the core samples to determine glucose and pentose yield. CJR designed and produced the core sampling device. KJHvM produced the core sampling device movie. SMK and NDL contributed to the design of the device and the study, and participated in data analysis and manuscript preparation. All authors read and approved the final manuscript.

## Supplementary Material

Additional 1 Core sampling device and sampling procedure movie. Click here for file

Additional file 2 Core sampling device computer-aided design (CAD). Click here for file

Additional file 3 **Table S1.** Analysis of variance for cell wall glucose yield (%) within internode sampling position. The second elongated internode above-ground was sampled in three plants of four maize inbred lines NK794, MS116, DKPB80 and A208 at three positions: bottom, middle and upper section. The analysis of variance was performed on the mean of the three plants. The error term is the genotype x position interaction. Click here for file

Additional file 4 **Table S2.** Analysis of variance for cell wall pentose yield (%) within internode sampling position. The second elongated internode above-ground was sampled in three plants of four maize inbred lines NK794, MS116, DKPB80 and A208 at three positions: bottom, middle and upper section. The analysis of variance was performed on the mean of the three plants. The error term is the genotype x position interaction. Click here for file

Additional file 5 **Table S3.** Analysis of variance for cell wall glucose yield (%) of stalk internode conserved at 4°C. The second elongated internode above-ground was sampled in three plants of five maize inbreds lines A305, A634, B85, SD102 and W59E and stored at ~4°C during 0, 24 and 60 hours. The analysis of variance was performed on the mean of the three plants. The error term is the genotype x hours of storage at 4°C interaction. Click here for file
