# Supplementary material for: A high-throughput core sampling device for the evaluation of maize stalk composition
Source: Biotechnol Biofuels. 2012 May 1;5:27. doi: 10.1186/1754-6834-5-27 (PMC3403939; doi:10.1186/1754-6834-5-27)
Supplement: Additional file 3 — Table S1. Analysis of variance for cell wall glucose yield (%) within internode sampling position. The second elongated internode above-ground was sampled in three plants of four maize inbred lines NK794, MS116, DKPB80 and A208 at three positions: bottom, middle and upper section. The analysis of variance was performed on the mean of the three plants. The error term is the genotype x position interaction. [file 1754-6834-5-27-S3.DOC]

**Additional file 3** - **Analysis of variance for cell wall glucose yield (%) within internode sampling position.** The second elongated internode above-ground was sampled in three plants of four maize inbred lines NK794, MS116, DKPB80 and A208 at three positions: bottom, middle and upper section. The analysis of variance was performed on the mean of the three plants. The error term is the genotype x position interaction.

| **Source of variation** | **df** | **Mean square** | **F-value** | **p-value** |
| --- | --- | --- | --- | --- |
| Genotype | 3 | 4.04 | 12.68 | 0.005 |
| Position | 2 | 1.24 | 3.88 | 0.083 |
| Error | 6 | 0.32 | - | - |

df, degrees of freedom
