# Supplementary material for: A high-throughput core sampling device for the evaluation of maize stalk composition
Source: Biotechnol Biofuels. 2012 May 1;5:27. doi: 10.1186/1754-6834-5-27 (PMC3403939; doi:10.1186/1754-6834-5-27)
Supplement: Additional file 5 — Table S3. Analysis of variance for cell wall glucose yield (%) of stalk internode conserved at 4°C. The second elongated internode above-ground was sampled in three plants of five maize inbreds lines A305, A634, B85, SD102 and W59E and stored at ~4°C during 0, 24 and 60 hours. The analysis of variance was performed on the mean of the three plants. The error term is the genotype x hours of storage at 4°C interaction. [file 1754-6834-5-27-S5.DOC]

**Additional file 5** - **Analysis of variance for cell wall glucose yield (%) of stalk internode stored at 4°C.** The second elongated internode above-ground was sampled in three plants of five maize inbreds lines A305, A634, B85, SD102 and W59E and stored at ~4°C during 0, 24 and 60 hours. The analysis of variance was performed on the mean of the three plants. The error term is the genotype x hours of storage at 4°C interaction.

| **Source of variation** | **df** | **Mean square** | **F value** | **p-value** |
| --- | --- | --- | --- | --- |
| Genotype | 4 | 27.59 | 86.41 | <0.001 |
| Hours of storage at 4°C | 2 | 0.32 | 1.00 | 0.41 |
| Error | 8 | 0.32 | - | - |

df: degrees of freedom.
